# Biopolymer and Carnauba Wax Coating for Improved Paper Oxygen Barrier, Water Vapor Barrier, and Grease Resistance

**DOI:** 10.1021/acsami.5c09638

**Published:** 2025-09-19

**Authors:** Sarah G. Fisher, Maya D. Montemayor, Alexandra Moran, Chiemeka Uwalaka, Jaime C. Grunlan

**Affiliations:** 14736 Texas A&M University, 400 Bizzell St., College Station, Texas 77840, United States

**Keywords:** biopolymer, carnauba wax, barrier coating, paper, food packaging

## Abstract

Food packaging is essential to avoid food waste, which is a major contributor to global emissions. The vast majority of food packaging materials are made from petroleum-derived plastic, contributing substantially to the plastic waste crisis. Cellulosic paper is a biobased and biodegradable alternative packaging material, but its use is limited by its extremely poor barrier to oxygen, water, water vapor, and grease, all of which contribute to early degradation of food products. Typical coatings for paper are unsustainable and inhibit its biodegradability. In this work, a water-based, fully bioderived polyelectrolyte complex coating consisting of chitosan, pectin, and carnauba wax is applied to kraft paper to improve its resistance to oxygen, water, and grease. After a thermal annealing step, the coatings exhibit continuous phases of wax and polyelectrolytes, resulting in a combination of both oxygen and water resistance. The best-performing coating substantially improves oxygen transmission rate (from 12.5 million to 155,000 cm^3^ m^–2^ day^–1^), water vapor barrier (from 346 to 304 g m^–2^ day^–1^), water absorptivity (from 131.6 to 29.1 g m^–2^) and castor oil absorptivity (from 94 to 53 g m^–2^), with minimal coat weight. This work advances the development of fully sustainable food packaging to address the major environmental crises of plastic and food waste.

## Graphical abstract

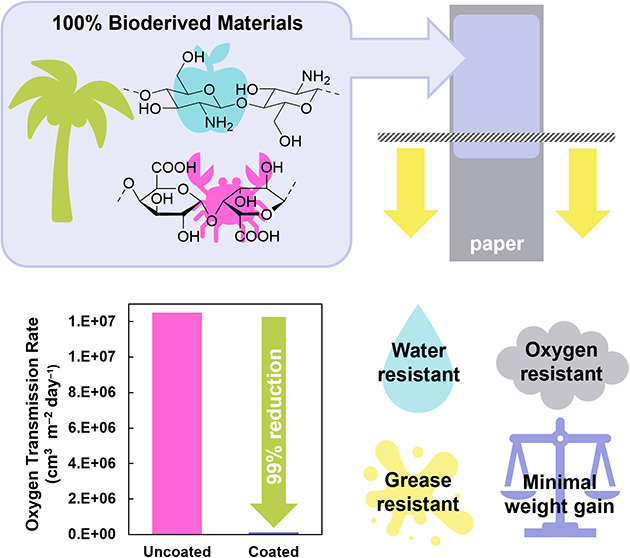

## Introduction

Plastic packaging accounts for over 30% of global plastic production, and more than half of the plastic packaging generated is used only once before being discarded.^1^ After use, about 80% of plastic waste accumulates in landfills and the environment, with approximately 8 million tons of plastic entering the ocean each year.^1,2^ In addition to harming aquatic and terrestrial life, the accumulation of plastic in the environment has resulted in increased levels of microplastics being detected across the food chain, including in most human organs, with the full health impacts currently unknown.^3–5^ In addition to causing environmental harm at its end of life, over 99% of plastic packaging is synthesized from petroleum-derived monomers, giving the packaging industry an equivalent annual oil consumption to the entire aviation industry.^1,6^

Proposed solutions to the plastic crisis include the well-known “3 Rs”: reducing the use of plastic, reusing plastic products as often as possible, and recycling plastic at the end of its life.^2^ It should be noted that these options are not always feasible for plastic packaging. Single use packaging prevents food waste, which is a major contributor to carbon dioxide in the atmosphere.^7^ Food packaging, which almost always consists of petroleum-derived plastic, extends the lifetime of food by protecting it from oxygen and water vapor that will cause early spoilage.^8,9^ The option to recycle plastic food packaging after use is attractive, but in reality, less than one tenth of plastic waste is recycled.^1,2^ This low level of recycling is due to lack of economic feasibility and the loss of desirable material properties. Additionally, it is impossible to recycle many blended and multilayered materials that are common in food packaging.^2,10^

Concerns regarding plastic packaging have led to a strong push toward more renewable packaging alternatives that come from renewable feedstocks and are biodegradable.^1,2,11,12^ One such material is paper. Made of interlaced cellulose fibers sourced from various plants, paper is printable, recyclable, biobased, and biodegradable.^9,12^ About 30% of packaging materials used today are constructed of paper and paperboard,^12^ but paper alone is not sufficient for food packaging because of its porous and superhydrophilic nature. As such, paper provides a poor barrier against gases, water, and grease. Paper’s barrier performance is commonly improved with the deposition of surface coatings.^9,12,13^ Coatings are typically synthetic petroleum-based polymers, or metallized layers, which inhibit the biodegradability and reprocessability inherent to paper.^12,13^ As such, there is a growing interest in the use of bioderived materials as coatings to improve the barrier properties of paper toward sustainable food packaging options.^12,14–17^ Individual biopolymers such as chitosan and pectin have been applied to paper and other substrates to improve the barrier performance, but it is generally observed that combining multiple biopolymers with favorable interactions leads to improved barrier.^18,19^

Polyelectrolyte complex (PEC) coatings have been applied to paper and other substrates as environmentally benign barrier layers.^20–23^ PEC coatings, which are prepared from polyelectrolytes of opposite charge, tend to exhibit an exceptional barrier to oxygen due to the high degree of ionic interpolymer cross-links in the material, leading to high cohesive energy density and low free volume for penetrants to infiltrate.^23^ For example, a PEC coacervate coating applied to paper reduced its oxygen transmission rate by 99%.^20^ A biobased PEC consisting of chitosan and pectin reduced the oxygen transmission rate of poly­(ethylene terephthalate) by 97% at about 1.5 μm of thickness.^24^ Despite their effective oxygen barrier, PEC coatings typically suffer from poor water resistance due to the inherent hydrophilicity of the polyelectrolyte components.^21,23^ In the past, layer-by-layer (LbL) assembled PEC coatings have been made hydrophobic via the incorporation of wax particles. For example, Glinel and co-workers deposited an anionic paraffin wax layer on top of a poly­(allylamine hydrochloride)/poly­(styrenesulfonate) layer-by-layer film in order to prevent water sorption.^25^ While the wax layer alone reduced diffusion substantially, an additional thermal treatment step to fuse the wax particles together into a continuous layer resulted in complete prevention of water diffusion. Another work reported a biobased layer-by-layer coating consisting of chitosan and carnauba nanoparticles applied to various fabrics to increase their hydrophobicity.^26^ Other, non-LbL multilayered coatings of polyelectrolytes and wax have also been developed for oxygen, water, and grease barrier performance.^27,28^ Despite these good results, the application of multilayered coatings is limited due to the large number of processing steps. “One-pot” buffer-cured PEC coatings have been developed that achieve comparable barrier performance to LbL films with few processing steps, where all the coating components are combined in a single solution or dispersion that is applied in one coating step.^21,29^ To the best of our knowledge, oil or wax particles have never been incorporated into a buffer-cured PEC coating, biobased or otherwise. Incorporating a biobased wax into a fully biobased buffer-cured PEC could allow for the development of a sustainable barrier coating with resistance to oxygen, water, and grease, that could be deposited with very few processing steps.

Naturally occurring polysaccharides, such as chitosan and pectin, are highly hydrophilic and cannot provide a barrier to water vapor or oxygen alone.^18^ Cross-linking, crystallization, or modification is typically needed to obtain desirable barrier performance.^19^ Such biopolymers can be utilized to form PEC coatings, which demonstrate excellent oxygen barrier performance due to ionic cross-linking but suffer from water sensitivity.^20,21,24^ Synthetic or naturally occurring waxes have been incorporated into biopolymer-based barrier coatings as well as synthetic polyelectrolyte layer-by-layer coatings for improved water resistance, but the oxygen barrier of such coatings is never reported, and grease resistance is rarely investigated.^25,26,28^ The combination of these properties in a single coating layer is quite promising for the future of sustainable food packaging.

Herein, a fully biobased PEC coating is developed utilizing chitosan, pectin, and carnauba wax with the goal of improving the barrier properties of kraft paper for sustainable food packaging. When applied to paper, the coating substantially improves its water resistance and oxygen barrier. This work combines for the first time the superior oxygen barrier properties of PEC coatings with the water resistance enabled by wax coatings, without requiring multiple coating layers or a heavy coat weight. Furthermore, the barrier to oxygen, water, and grease are all thoroughly investigated toward an application in food packaging where all these properties are required. This low weight coating also shows moderate resistance to grease and castor oil. This work is believed to be the first time wax particles have been incorporated into a buffer-cured polyelectrolyte complex coating, and the first time the grease resistance of such coatings has been evaluated. This fully sustainable paper barrier system represents a substantial development in addressing the plastic waste crisis by providing a renewable alternative to conventional food packaging materials.

## Experimental Section

### Materials and Substrates

Pectin from apple (PC), citric acid monohydrate, sodium hydroxide (NaOH, ACS reagent, pellets), heptane (anhydrous, 99%), and castor oil were purchased from Sigma-Aldrich (St. Louis, MO). Chitosan (CH, 95% deacetylated) was purchased from Greentech Biochemicals (Qingdao, China). AquaBead 425E anionic carnauba wax (CW) emulsion was purchased from Micro Powders, Inc. (Tarrytown, NY). Hydrochloric acid (HCl, 5.0 N) was purchased from VWR International (Radnor, PA). Toluene (ACS reagent) was purchased from Fisher Chemical (Pittsburgh, PA). Kraft builder’s paper (TRIMACO Easy Mask, 10 mil, 106 g m^–2^) was purchased from Home Depot (College Station, TX) and used as the substrate for coat weight, scanning electron microscopy, oxygen transmission rate, kit test, Cobb test, castor oil Cobb test, water contact angle, water vapor transmission rate, and dynamic mechanical analysis experiments. Single-side polished, 500 μm-thick Si wafers were purchased from University Wafer (Boston, MA) and used as substrates for thickness measurements, FTIR, atomic force microscopy, and water contact angle tests. Eighteen MΩ deionized (DI) water was used to prepare all solutions and dispersions and for all rinsing procedures.

### Coating Preparation

Aqueous PC solutions were prepared and stirred overnight. CW emulsion was added to the PC solution to result in a coating dispersion with a final concentration of 3% (w/w) PC and 0, 0.5, or 1% CW. The PC (+CW) dispersion was adjusted to pH 2.2 using 5 M HCl. An aqueous 1% CH solution was prepared and adjusted to pH 1.5 using 5 M HCl. Equal volumes of PC (+CW) dispersion and CH solution were combined and stirred overnight to form the coating dispersion.

### Coating Deposition

Prior to coating, silicon wafers were washed with DI water, methanol, then DI water again, dried with compressed air, and plasma treated for 5 min to improve coating adhesion. Paper was not washed or pretreated prior to coating. PEC coatings were deposited on one side of the substrate using a #30 wire-wound Mayer rod applicator (Gardco, Columbia, MD). Coated samples were allowed to dry flat at room temperature before being dipped in a pH 5 200 mM citric acid buffer for 5 min to cure. After curing, the samples were rinsed in DI water three times for 30 s each, then dried flat at room temperature. Thermal annealing was then performed at 90 °C for 1 h before the samples were slowly cooled back to room temperature.

### Characterization

Coating thickness was measured using a P6 profilometer (KLA-Tencor, Milpitas, CA). An average of six measurements was reported. Coat weight and weight gain were measured by punching a 1″ diameter circle of coated paper and taking the mass with an analytical balance. An average of five measurements was reported. The coat weight was calculated by subtracting the mass of an uncoated paper circle (in g) from the mass of a coated paper circle and dividing by the area of the circle (in m^2^) (i.e., *W* = (*M*_coated_ – *M*_uncoated_)/*A*). The weight gain was calculated by dividing the mass increase with the coating by the average mass of five uncoated paper samples (i.e., WG = (*M*_coated_ – *M*_uncoated_)/*M*_uncoated_). FTIR spectroscopy was performed using an Alpha Platinum-ATR FTIR spectrometer (Bruker Optics Inc. Billerica, MA), with air as a background. A minimum of 24 scans were signal averaged for each sample and the scan resolution was 4 cm^–1^. Scanning electron microscope (SEM) images of coated and uncoated paper were obtained using a TESCAN Vega 3 scanning electron microscope (Brno, Czech Republic) after sputter coating with 5 nm of gold. A Bruker Dimension Icon atomic force microscope [AFM] (Billerica, MA) was used to evaluate the surface morphology and phase behavior of the coatings. Static water contact angles were measured using a KSV CAM 200 instrument (KSV Instruments, Ltd., Monroe, CT). Data was recorded 30 s after depositing a 5 μL DI water droplet on the surface of coated or uncoated substrate. An average of 10 data points was recorded for each sample. Water absorptiveness was measured via the Cobb_60_ test in accordance with ISO 535:2023. Castor oil absorptiveness was measured via the same test parameters (referred to as the oil Cobb test), using castor oil instead of water. Water vapor transmission rate was measured in triplicate according to ASTM E96/E96M–24 using the water method, with the samples having the coated side up, at 30 °C and an average of 50% relative humidity. The test cup was composed of acrylonitrile butadiene styrene (ABS) and the gaskets were composed of thermoplastic polyurethane (TPU). Each run lasted 3 h, and the region of the curve corresponding to the final 2 h was used to calculate the water vapor transmission rate. Grease resistance was measured according to TAPPI T 559. Oxygen transmission rate (OTR) was performed by MOCON Inc. (Minneapolis, MN) using an OpTech-O2 model P instrument. OTR testing utilized room air (20.9% O_2_) as a test gas at 23.0 °C, with a relative humidity (RH) of 39%, in accordance with ASTM F3136. Dynamic mechanical analysis (DMA) tensile test experiments were carried out with a TA Instruments DMA 850 (McHenry, IL), using samples of ∼30 mm by ∼2.5 mm. Each measurement was carried out at a strain ramp rate of 2.5% min^–1^ and a 10 mm gauge length until break at ambient conditions. Five trials were performed for each sample.

## Results and Discussion

### Structure and Bonding of Polyelectrolyte Complex Coatings

Aqueous coating dispersions consisting of 1.5 wt % pectin (PC) and 0.5 wt % chitosan (CH) were prepared with concentrations of carnauba wax (CW) varying from 0 to 0.5 wt %. PC has a p*K*_a_ of ∼2.8 to 4.1,^30^ while CH has a p*K*_a_ of ∼6.5.^31^ Coating dispersions were prepared at a pH of ∼2, where the carboxylic acid groups of PC are protonated, and therefore the polyelectrolyte is uncharged. This allows CH, which is positively charged at pH 2, and PC to coexist in solution without forming a solid complex. After the coating dispersion is deposited, the coated substrates were immersed in a citric acid (CA) buffer at a pH of 5. At pH 5, at least 50% of the carboxyl groups in PC are expected to be deprotonated, resulting in the formation of ionic cross-links with chitosan.^30,32^ The ionic cross-linking process imparts high gas barrier behavior by increasing the cohesive energy density and minimizing free volume in the coating.^21,33^ After buffer curing, some of the samples were subjected to a 60 min thermal annealing step above the melting temperature of the wax. The goal of this step was to melt the CW particles to form a continuous wax layer, as this was predicted to result in maximal hydrophobicity of the coating based on previous work.^25^ The coating process is summarized in Figure 1A. Figure 1B shows the structures of the coating components. Figure 1C shows schematics of the four possible combinations of film postprocessing: uncured and not annealed, cured and not annealed, uncured and annealed, and cured and annealed. Coatings with 0 wt % CW, 0.25 wt % CW, and 0.5 wt % CW were individually prepared using each of these four processes, resulting in a total of 12 unique coatings for analysis, as shown in Table 1. The coatings are referred to by their CW content.

**Figure 1:**
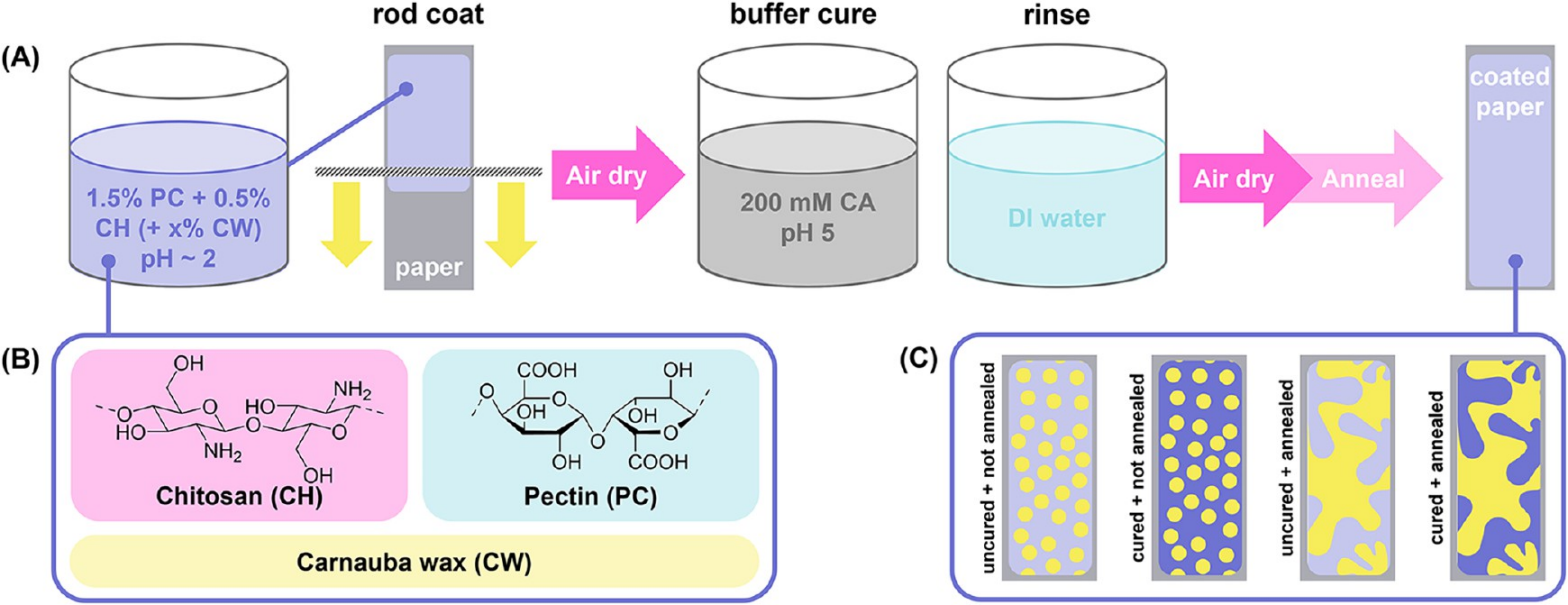
(A) Polyelectrolyte complex coating process, (B) coating components, and (C) schematics of films with and without buffer curing and thermal annealing.

**Table 1:** Summary of Coating Formulations Tested

wax concentration in coating dispersion	theoretical wax concentration in dried coating^a^	buffer cured?	annealed?
0 wt %	0 wt %	No	No
		Yes	No
		No	Yes
		Yes	Yes
0.25 wt %	11 wt %	No	No
		Yes	No
		No	Yes
		Yes	Yes
0.5 wt %	20 wt %	No	No
		Yes	No
		No	Yes
		Yes	Yes

a Wax solids divided by total solids of coating dispersion.

Buffer-induced ionic cross-linking was confirmed via Fourier-transform infrared (FTIR) spectroscopy, as shown in Figure 2. The uncured 0% CW coating exhibits a sharp peak at 1736 cm^–1^ corresponding to the carbonyl stretch of PC. After curing, the carbonyl stretching peak decreases in intensity and asymmetric and symmetric carboxylate stretching peaks appear at 1575 and 1400 cm^–1^, respectively, indicating that PC has been deprotonated. In addition, the broad hydroxyl stretch at 3300 cm^–1^ is decreased in intensity, which is commonly observed upon buffer-curing PECs due to a loss of water upon ionic cross-linking.^21,24^ Upon incorporation of 0.5% CW, two sharp absorbance peaks appear at 2916 and 2850 cm^–1^. These peaks are attributed to the asymmetric and symmetric stretching modes of aliphatic hydrocarbons in CW and indicate successful incorporation of wax in the coating.^34^ Upon curing, the 0.5% CW system shows the same appearance of carboxylate stretches and decrease in carbonyl and hydroxyl stretches seen in the 0% CW system, indicating that the presence of CW in the coating does not impede the curing process. Figure S1 shows FTIR spectra of coating components and all coating recipes prepared. The 0.25% CW coatings show the same trend as 0.5% CW, with less intense aliphatic hydrocarbon peaks at 2916 and 2850 cm^–1^ due to the lower wax content. Thermal annealing does not appear to change the chemical structure of either the individual coating components (Figure S1A) or any of the coating formulations (Figure S1B).

**Figure 2:**
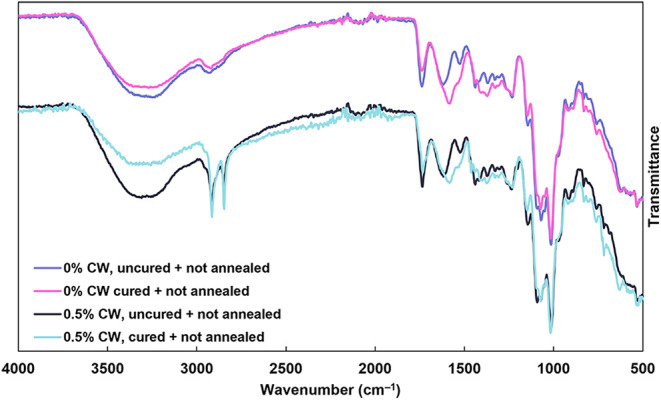
FTIR spectra of cured and uncured coatings with 0% CW and 0.5% CW. FTIR spectra of coating components and of all recipes, with and without curing and annealing, are shown in Figure S1.

Coated paper is visually indistinguishable from uncoated paper due to the relatively low coat weight (Figure S2). The coating morphology was imaged using scanning electron microscopy (SEM). As shown in Figure 3, uncoated paper is fibrous and highly porous, which contributes to its poor barrier against gases, water, and grease.^9,12,13^ The coating is relatively conformal to the paper structure, preserving the individual fibers, but appears to fill in major pores between the fibers to improve barrier performance. No visual difference is observed between uncured and buffer-cured samples. Wax particles are visually evident in the 0.25% and 0.5% CW coatings. It is apparent that fewer particles are observed after annealing, which is likely due to individual particles melting and fusing during the annealing process. This phenomenon has been previously reported for thermally annealed carnauba wax coatings on paper.^35^

**Figure 3:**
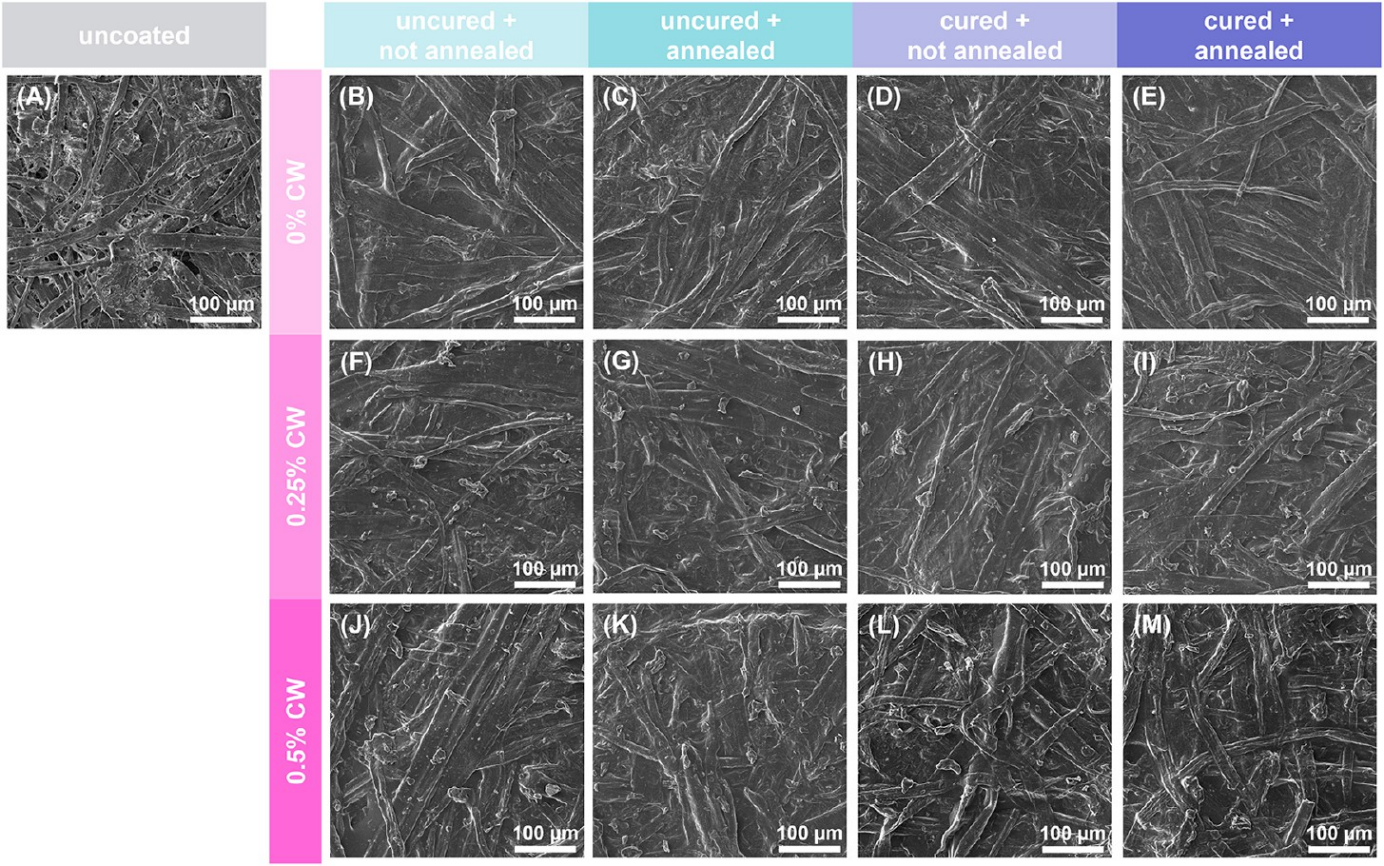
SEM images of (A) uncoated paper and paper coated with (B–E) 0% CW coatings, (F–I) 0.25% CW coatings, and (J–M) 0.5% CW coatings: (B, C, F, G, J, K) uncured and (D, E, H, I, L, M) buffer cured, and (B, D, F, H, J, L) not annealed and (C, E, G, I, K, M) thermally annealed.

Coatings were thermally annealed at 90 °C, above the melting temperature of CW.^36^ The purpose of this treatment was to melt the wax particles, fusing them together to form a continuous wax layer that was expected to improve water barrier. This process was monitored using atomic force microscopy (AFM) phase mapping images. As shown in Figure 4, PEC coatings with no wax exist as single-phase materials due to the intricate blending of the two polyelectrolyte components.^23,37^ CW-containing coatings before annealing look very similar to the coatings with no wax. Upon thermally annealing the wax-containing coatings, a dramatic change in phase behavior is observed. Thermally cured coatings exhibit two distinct phases of polyelectrolytes and wax, which appear to be cocontinuous throughout the coating. Figure S3 demonstrates the gradual formation of this cocontinuous network as a function of annealing time. Because there is little difference between 45 and 60 min of annealing time, it is assumed that complete annealing is achieved at 60 min, so this time was selected as the annealing time for all samples. The formation of a cocontinuous two-phase material was expected to optimize combined oxygen and water barrier performance, as CH/PC PEC coatings have already demonstrated good oxygen barrier properties, while carnauba wax imparts hydrophobicity.^24^

**Figure 4:**
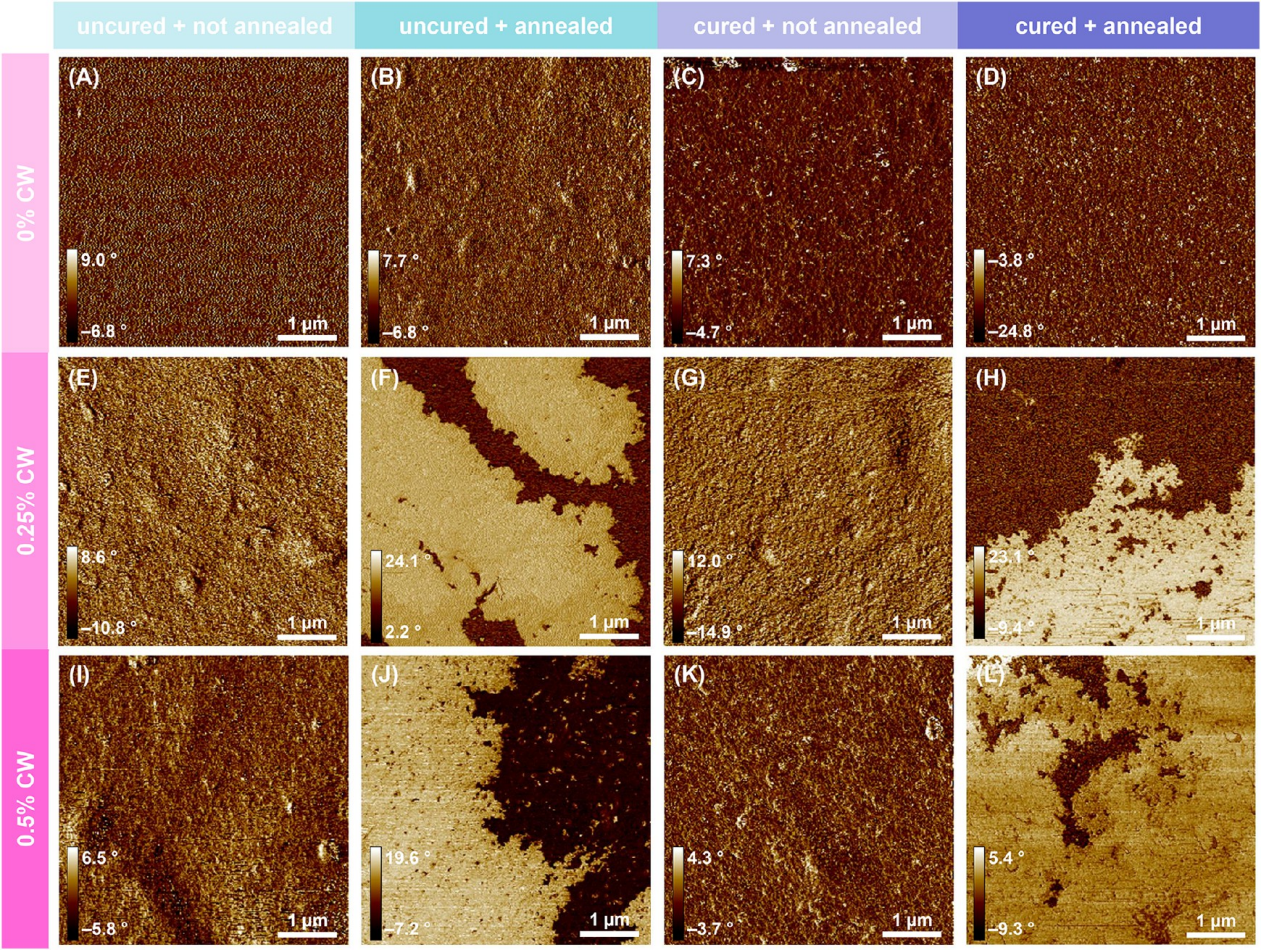
AFM phase mapping images of (A–D) 0% CW coatings, (E–H) 0.25% CW coatings, and (I–L) 0.5% CW coatings: (A, B, E, F, I, J) uncured and (C, D, G, H, K, L) buffer cured, and (A, C, E, G, I, K) not annealed and (B, D, F, H, J, L) thermally annealed.

### Water Barrier

Static water contact angle measurements were made in order to evaluate the influence of the coating on the substrate’s hydrophilicity. Thermally annealed carnauba wax coatings have been shown to increase the contact angle of paper.^35^ As previously discussed, uncoated paper is by nature hydrophilic, and it is not possible to make a contact angle measurement on it because the water droplet is instantly absorbed (i.e., the contact angle is effectively 0°). In contrast, the PEC coatings provide a substantial improvement to the water sensitivity of paper. Contact angle measurements were made on both coated paper and coated silicon wafers. When applied to paper, all the coating formulations result in hydrophobic behavior, with contact angles greater than 90° (Figure S4). Figure 5 shows representative photographs of the water contact angle performance of uncoated and coated paper. It is apparent from the photographs that uncoated paper is fully wetted by the water droplet after 30s, while coated paper resists absorption of the water droplet for much longer. No clear trend is observed in terms of buffer curing, annealing, or wax concentration. Because contact angle is also heavily dependent on surface morphology,^38^ coatings on silicon wafers were also evaluated to better understand the effect of coating formulation without the influence of substrate roughness.

**Figure 5:**
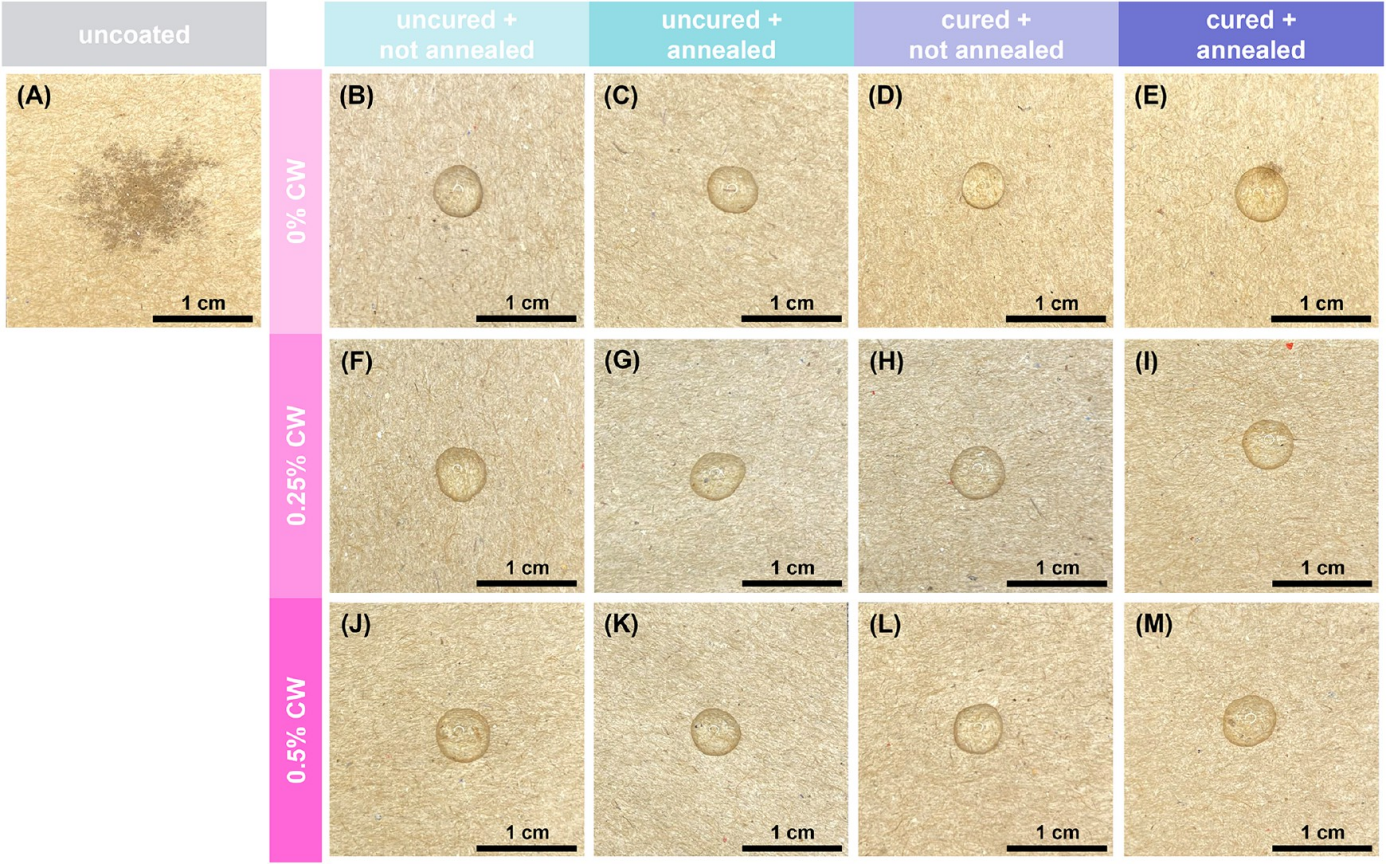
Representative photographs of water droplets on paper: (A) uncoated paper, paper coated with (B–E) 0% CW coatings, (F–I) 0.25% CW coatings, and (J–M) 0.5% CW coatings: (B, C, F, G, J, K) uncured and (D, E, H, I, L, M) buffer cured, and (B, D, F, H, J, L) not annealed and (C, E, G, I, K, M) thermally annealed. Photos taken 30 s after contact with water droplet.

When measured on silicon wafer substrates, the influence of wax concentration, buffer curing, and annealing are far more clear. As shown in Figure 6A, the coatings with no wax exhibit hydrophilic contact angles of 46–59°. While there is no significant change in contact angle for the uncured 0% CW coating upon annealing, there is a significant difference between uncured and cured samples (regardless of annealing), with the cured samples exhibiting a higher contact angle (i.e., they are less hydrophilic). A decrease in water sensitivity/hydrophilicity upon buffer curing is commonly observed for PECs and is attributed to the formation of a strong network of ionic cross-links increasing cohesive energy in the film.^21,23^ Interestingly, there is also a slight increase in contact angle upon annealing, even though no wax is present in the coating to be melted and rehardened into a hydrophobic layer. This slight increase could be attributed to thermally triggered covalent cross-linking between the oppositely charged polyelectrolytes, which has been demonstrated in the past to improve water resistance.^33,39^

**Figure 6:**
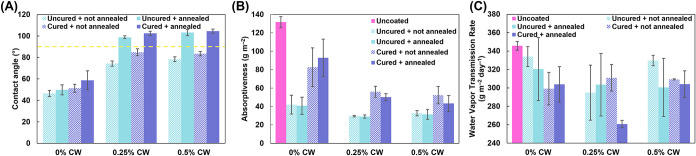
(A) Water contact angle of coatings on Si wafers (the yellow dotted line marks a contact angle of 90°), (B) Cobb_60_ water absorptiveness of uncoated and coated paper, and (C) water vapor transmission rate of uncoated and coated paper. All results are shown in Table S2.

When wax is incorporated into the PEC, the contact angle increases significantly whether or not samples are cured or annealed. It is apparent for the 0.25% CW and 0.5% CW samples that independent increases in hydrophobicity occur upon buffer curing and annealing, with the highest contact angles (over 100°) being achieved when samples are both buffer cured and annealed. As in the 0% CW samples, the increase in contact angle with curing is attributed to ionic cross-linking. The increase in contact angle with annealing is primarily attributed to the formation of a continuous wax network within the coating, which is more effective at blocking water penetration than simply including wax nanoparticles in the coating because it covers the entire surface. The increase in contact angle upon annealing may also be partially due to thermal cross-linking, but this is unlikely to be the full explanation, as the increase in contact angle with annealing is much greater in the wax-containing samples than in the 0% CW samples.

Water resistance of uncoated and coated paper was further evaluated with a Cobb_60_ water absorptiveness test, which measures the ability of a sample to resist the absorption of liquid water. As shown in Figure 6B, uncoated paper has a water absorbance value of 132 g m^–2^. All the PEC coatings significantly reduce the water absorbance of paper to values ranging from 29 to 93 g m^–2^, with wax-containing samples exhibiting lower water absorbance than their corresponding 0% CW samples. Regardless of wax content, the annealing process does not appear to have a significant influence on the water absorptiveness of coated paper. Interestingly, buffer curing appears to have an adverse effect on the water absorptiveness of coated paper. This does not follow the trend observed in contact angle measurements. It is believed that the buffer curing process, which involves soaking coated paper samples in an aqueous buffer solution for five minutes and rinsing with water, may compromise the integrity of the paper to some degree, rendering it more susceptible to water absorption. A similar phenomenon was observed in a previous work, where buffer curing of coated paper resulted in a loss of oxygen barrier performance attributed to loosening of the paper fibers.^20^ Regardless, all coatings substantially decrease amount of liquid water absorbed.

The water resistance of the coatings was further investigated by measuring the water vapor transmission rate (WVTR) of uncoated and coated paper. The results of the water vapor barrier tests are summarized in Figure 6C. It is important to note that paper is a natural material and therefore extremely heterogeneous, which can make it difficult to draw concrete conclusions about the influence of wax concentration, buffer curing, and thermal annealing on water vapor barrier. With that being said, it appears that the 0% CW coating with no buffer curing (regardless of annealing) does not substantially improve the water vapor barrier of uncoated paper, which is about 346 g m^–2^ day^–1^. Upon buffer curing (again, regardless of annealing), the water absorptiveness drops substantially. This is to be expected, as ionic cross-linking reduces the free volume in the coating, leading to better barrier performance.^21,23^ The incorporation of CW decreases the barrier of the uncured coatings to a comparable extent, regardless of whether the wax concentration is 0.25% or 0.5%. In the case of the 0.25% CW coating, buffer curing and annealing result in the lowest attained water vapor transmission rate of 261 g m^–2^ day^–1^, a ∼25% reduction from uncoated paper. No clear trend in WVTR with curing or annealing is observed for the 0.5% CW coatings. This may simply be due to the heterogeneous nature of the paper substrate. It is also likely that there are diminishing returns in water resistance beyond a particular wax concentration. When the water contact angles of coatings with up to 1.5% CW were measured, no change in contact angle was observed with increasing wax content beyond 0.5% (Figure S5). Unless otherwise noted, all water barrier tests were performed under ambient conditions (22 °C, 50% RH), as these are the conditions most relevant to the application.

### Oxygen Barrier

Oxygen barrier performance was evaluated by measuring the oxygen transmission rate (OTR) of uncoated and coated paper under ambient conditions. As shown in Figure 7, uncoated paper has an enormously high OTR of 12,500,000 cm^3^ m^–2^ day^–1^. This has been previously observed and is attributed to the extremely porous nature of paper.^20^ Coatings with 0% and 0.25% CW were evaluated, as little difference in oxygen barrier performance was expected between 0.25% and 0.5% CW coatings. Regardless of coating formulation, all coated paper samples exhibit a substantial reduction in OTR (>95%) relative to uncoated paper. Exact OTR values are listed in Figure 7. This substantial barrier improvement is attributed to the coating filling in pores in the paper substrate as well as the high cohesive energy density of the polyelectrolyte complex coating. When comparing between coating formulations, it can be observed that each cured system has a higher OTR than its respective uncured system. Buffer curing is expected to increase the charge density of the polyelectrolytes in the coating, leading to better barrier performance, but it is likely that the buffer curing process physically disrupts the weave of the paper substrate at a detriment to the barrier performance.^20^

**Figure 7:**
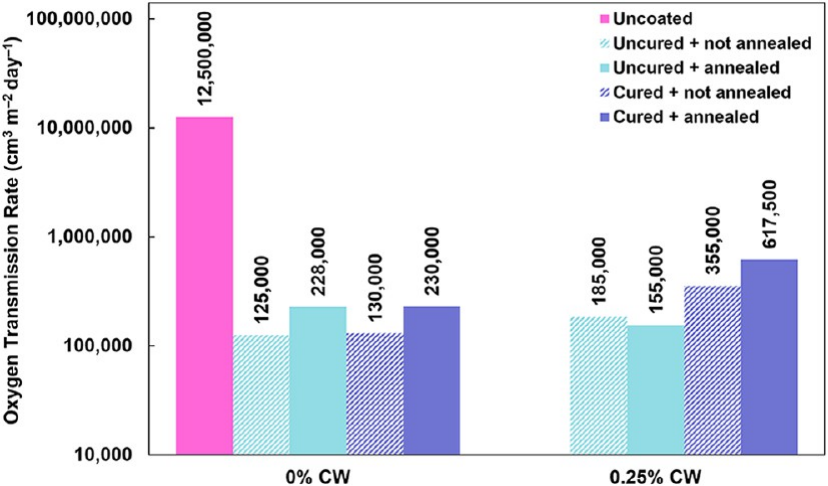
Oxygen transmission rate of uncoated and coated paper. Results are shown in Table S3.

Thermal annealing also appears to decrease the barrier performance (increase the OTR) for the 0% CW coatings, but this may simply be due to variations in the heterogeneous substrate. It also appears that incorporating wax into the PEC coatings decreases the barrier performance to some extent. Although carnauba wax can play a role in oxygen barrier,^40^ the slight increase in OTR with the addition of CW to the coating suggests that the oxygen barrier performance of the coatings comes primarily from the polyelectrolyte matrix rather than the wax. This is expected due to the high degree of ionic cross-links in the polyelectrolyte matrix, leading to very high cohesive energy density.^23^ The best-performing coating, 0% CW with no curing or annealing, has an OTR of 125,000 cm^3^ m^–2^ day^–1^, a 99% improvement over uncoated paper, with less than 5 wt % added. This represents a substantial improvement over a previously reported polyelectrolyte complex barrier coating for the same paper substrate, which had an OTR of 126,000 cm^3^ m^–2^ day^–1^ (still a 99% improvement), but required a double-coating with a weight gain of 40%.^20^ Coat weight and weight gains for all paper samples are listed in Table S1.

### Grease Barrier

As previously discussed, uncoated paper is a poor barrier to grease due to its porosity.^12,13^ Biobased grease barrier coatings typically function by a physical means rather than chemical, with the coating forming a barrier layer on the paper surface to resist grease by increasing the critical surface tension required for wettability.^41^ The oil absorptiveness of uncoated and coated paper was evaluated using the Cobb_60_ test (ISO 535:2023), using castor oil instead of water, with results shown in Figure 8A. As expected, uncoated paper displays a high oil absorptiveness value of 94 g m^–2^. All investigated coatings substantially decrease the oil absorptiveness to values ranging from 41 to 67 g m^–2^. Grease resistance was further evaluated with the kit test (TAPPI T 559), in which each sample receives a rating based on its ability to withstand increasingly aggressive grease solutions. In this test, a higher rating indicates greater grease resistance. Results of the kit test are shown in Figure 8B. Again, as expected, uncoated paper performs extremely poorly, with a rating of 0 (the lowest possible). As in the oil Cobb_60_ test, all coated samples performed significantly better, with intermediate kit values of 2 to 5. Figure 9 shows representative photos of the response of uncoated and coated paper to a droplet of castor oil after 15 s, corresponding to a kit rating of 1. The darkening of the uncoated paper upon contact with castor oil indicates failure of the level 1 test, while coated papers are not readily wetted by the castor oil droplet.

**Figure 8:**
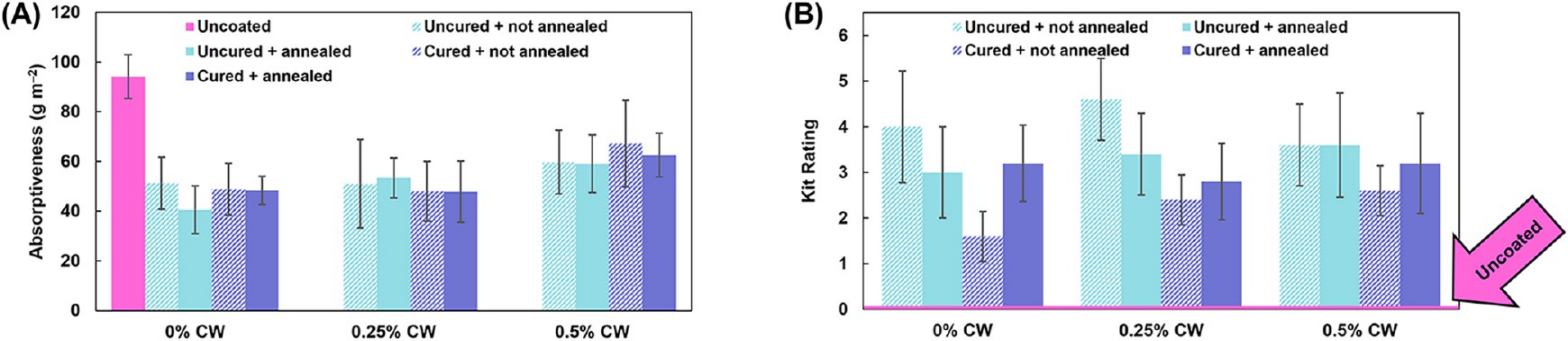
(A) Cobb_60_ castor oil absorptiveness of uncoated and coated paper and (B) kit grease resistance rating of uncoated and coated paper. All results are tabulated in Table S3.

**Figure 9:**
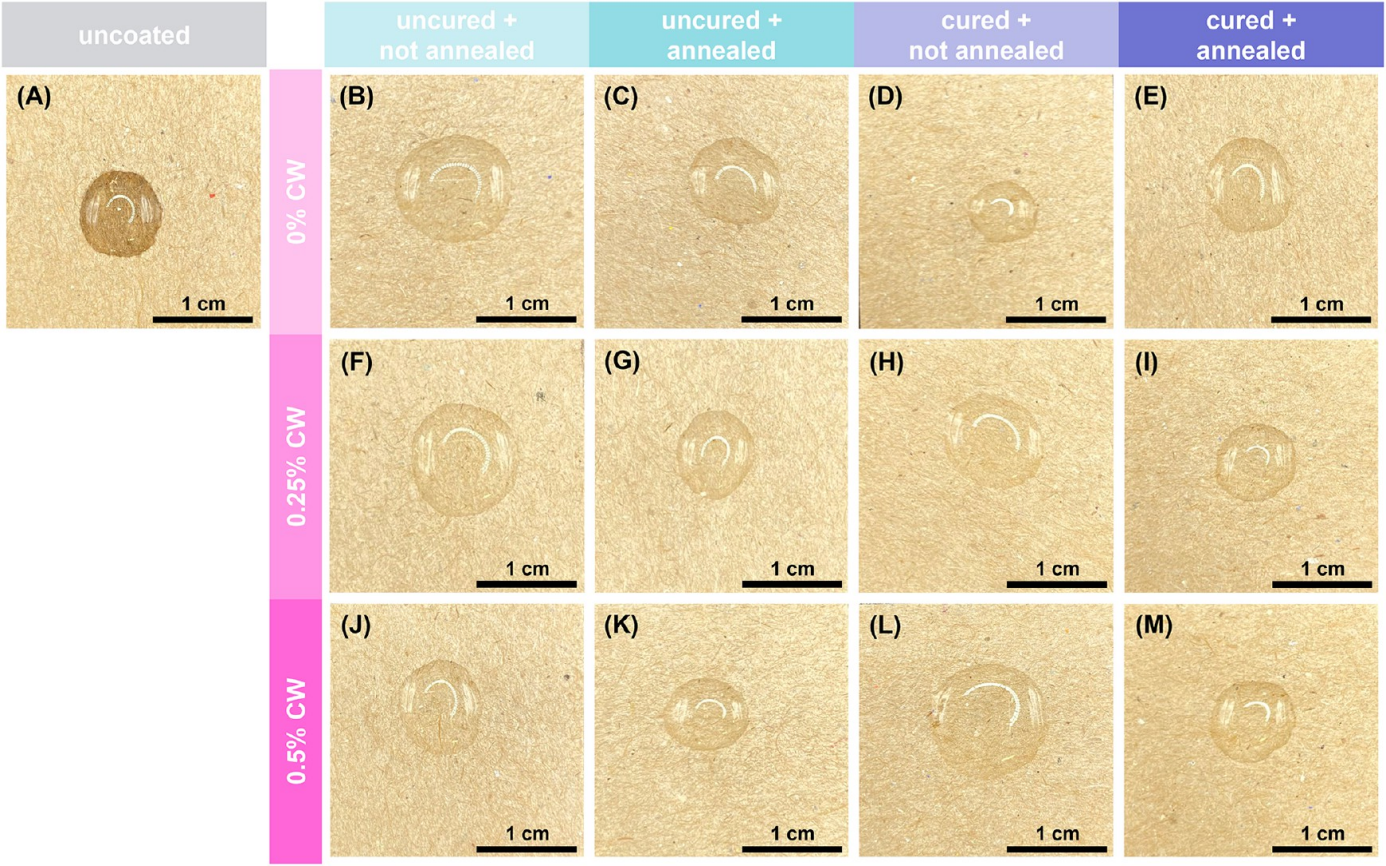
Representative photographs of castor oil droplets on paper: (A) uncoated paper, paper coated with (B–E) 0% CW coatings, (F–I) 0.25% CW coatings, and (J–M) 0.5% CW coatings: (B, C, F, G, J, K) uncured and (D, E, H, I, L, M) buffer cured, and (B, D, F, H, J, L) not annealed and (C, E, G, I, K, M) thermally annealed. Photos taken 15s after contact with oil droplet.

In both the oil Cobb_60_ test and the kit test, it is difficult to determine a trend in grease resistance with wax content, curing, or heating. This suggests that the efficacy of the coating at blocking grease is not due to any of these factors. Instead, it is likely that the coatings increase the grease resistance of paper by physically filling in the pores into which oil and grease are easily absorbed. The kit results also mirror what was observed in the water Cobb_60_ testing. Curing has a slight adverse effect on grease resistance, as it did for water resistance. Again, this supports the conclusion that the mechanism of grease barrier is physical rather than chemical, as buffer-induced ionic cross-linking is typically expected to decrease free volume in PEC coatings, leading to better barrier performance. In addition, if the mechanism was chemical, an inverse trend would be expected when comparing water absorption results to grease resistance (i.e., a hydrophobic versus a hydrophilic behavior). Instead, both results are worsened with curing, which supports the previously mentioned hypothesis that the buffer curing process compromises the paper (making it more susceptible to penetrants). Regardless, all coatings (including buffer-cured coatings) significantly improve the oil and grease barrier of uncoated paper. While the grease resistance obtained is relatively moderate, this study is believed to represent the first ever investigation of this behavior with a buffer-cured PEC coating. Future work is certainly needed to further improve the grease resistance of such coatings.

### Mechanical Properties

Figure S6 shows the results of tensile testing of uncoated and coated paper. Because of the heterogeneous nature of paper, relatively large variation is observed between individual samples. Even so, it can generally be seen that the presence of the coating does not significantly impact the elastic modulus or the tensile strength of paper. It is clear that the coating substantially improves the water, oxygen, and grease barrier of the paper without changing its desirable mechanical properties. Figure 10 shows an example of the intended application of coated paper as a sustainable food packaging material.

**Figure 10:**
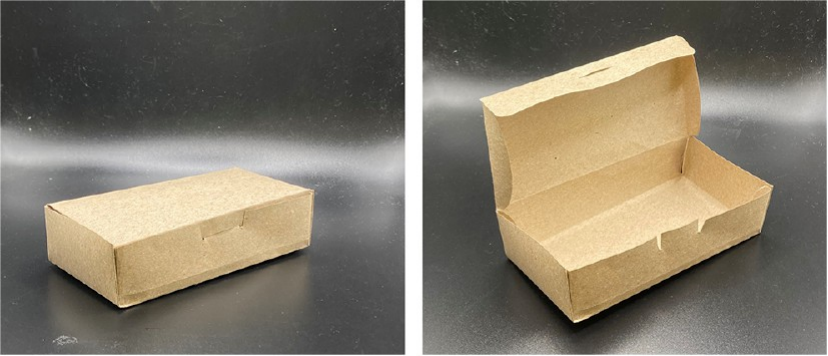
Box constructed from kraft paper as an example application of coated paper for food packaging.

## Conclusion

A biobased polyelectrolyte complex coating was applied to paper to improve its resistance to oxygen, water, and grease. Coatings were cured via a buffer step to induce ionic cross-linking by increasing the charge concentration of the oppositely charged polyelectrolytes in the coating. Carnauba wax particles were incorporated into the polyelectrolyte complex coating at varying concentrations to form a composite coating that, when thermally annealed, exhibited cocontinuous regions of polyelectrolyte and wax that resulted in combined oxygen and water resistance. The coating with the best combination of properties (0.25% CW uncured and annealed) decreased the oxygen transmission rate of paper from 12.5 million to 155,000 cm^3^ m^–2^ day^–1^, decreased the water vapor transmission rate from 346 to 304 g m^–2^ day^–1^, decreased the water absorptivity from 131.6 to 29.1 g m^–2^, and decreased the castor oil absorptivity from 94 to 53 g m^–2^, with less than 5% mass added relative to an uncoated paper substrate. This work represents a significant step toward the development of fully renewable food packaging materials.

## Supplementary Material



## Notes

FTIR spectra of coating components and all coating recipes (Figure S1); Photographs of uncoated and coated paper (Figure S2); AFM phase images of 0.5% CW coatings during the annealing process (Figure S3); Water contact angles of coatings on paper (Figure S4); Water contact angles of coatings on Si wafers (Figure S5); Mechanical properties of uncoated and coated paper (Figure S6); Coat weight and weight gain of coatings on paper (Table S1); Water response and water vapor barrier properties of uncoated and coated paper (Table S2); Oxygen and grease barrier properties of uncoated and coated paper (Table S3) (PDF)

## Abbreviations

PEC: polyelectrolyte complex
LbL: layer-by-layer assembly
PC: pectin
CH: chitosan
CW: carnauba wax
OTR: oxygen transmission rate
WVTR: water vapor transmission rate
