# Supplementary material for: Biopolymer and Carnauba Wax Coating for Improved Paper Oxygen Barrier, Water Vapor Barrier, and Grease Resistance
Source: ACS Appl Mater Interfaces. 2025 Sep 19;17(39):55307–17. doi: 10.1021/acsami.5c09638 (PMC12492330; doi:10.1021/acsami.5c09638)
Supplement: Supplementary file 1 [file am5c09638_si_001.pdf]

Supporting information for:

Biopolymer and Carnauba Wax Coating for Improved Paper Oxygen Barrier, Water Vapor Barrier, and Grease Resistance

Sarah G. Fisher, Maya D. Montemayor, Alexandra Moran, Chiemeka Uwalaka, Jaime C. Grunlan*

Texas A&M University, 400 Bizzell St., College Station, TX, 77840, USA

Corresponding author: Jaime C. Grunlan (jgrunlan@tamu.edu)

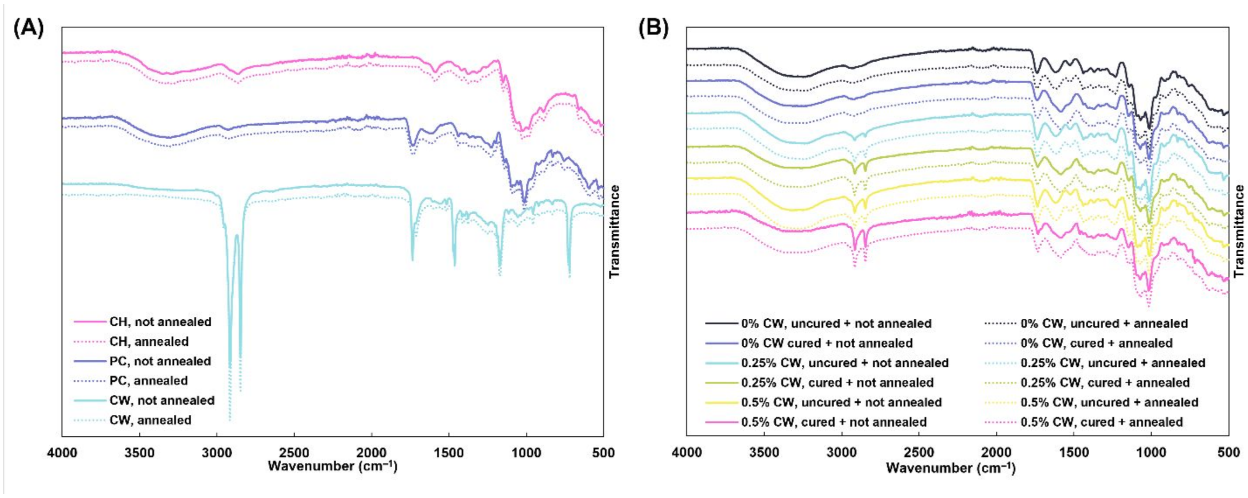

Figure S1. FTIR spectra of (A) coating components with and without thermal annealing, and (B) all coating recipes with and without curing and annealing.

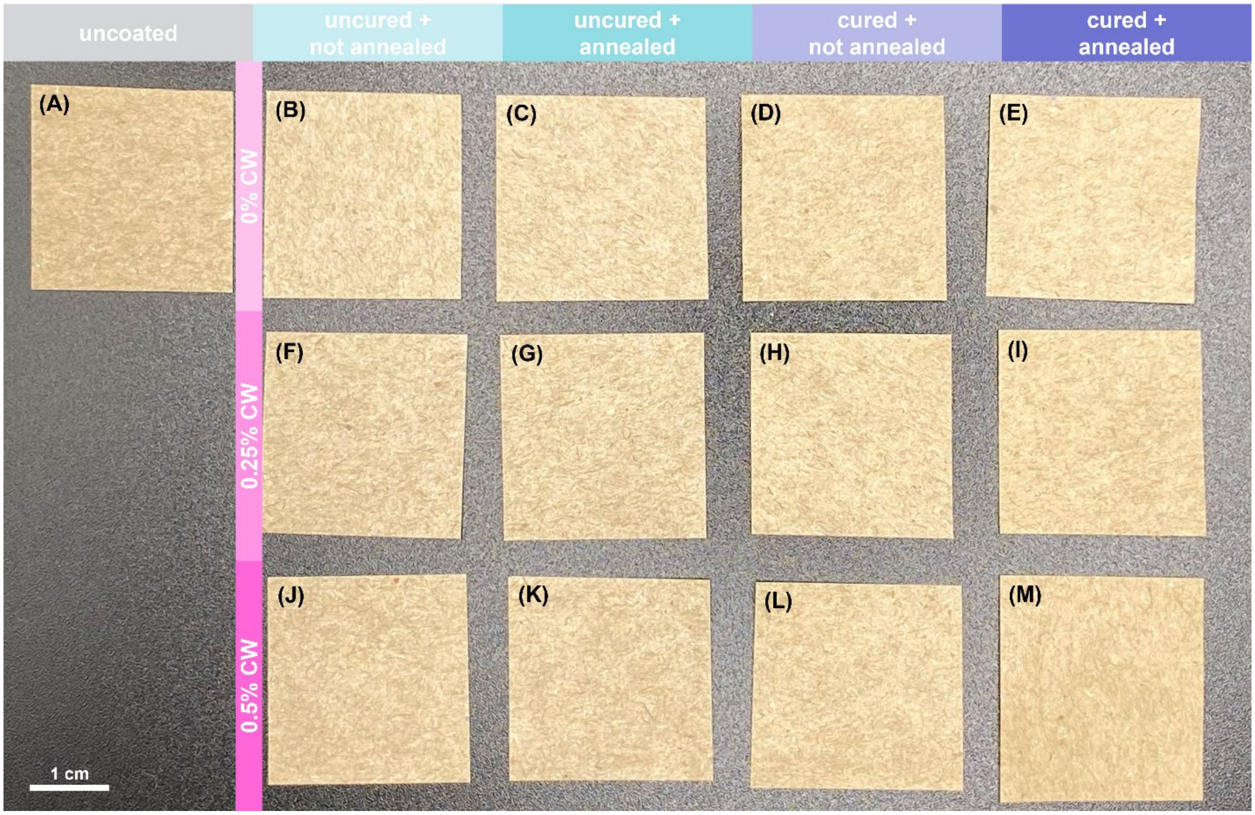

Figure S2. Photograph of (A) uncoated paper and paper coated with (B-E) 0% CW coatings, (F-I) 0.25% CW coatings, and (J-M) 0.5% CW coatings: (B,C,F,G,J,K) uncured and (D,E,H,I,L,M) buffer cured, and (B,D,F,H,J,L) not annealed and (C,E,G,I,K,M) thermally annealed.

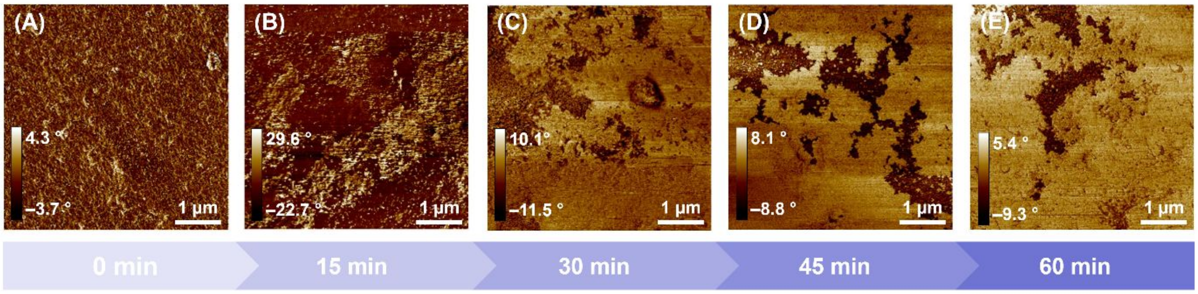

Figure S3. AFM phase mapping images of 0.5% CW coatings annealed for (A) 0 minutes, (B) 15 minutes, (C) 30 minutes, (D) 45 minutes, and (E) 60 minutes.

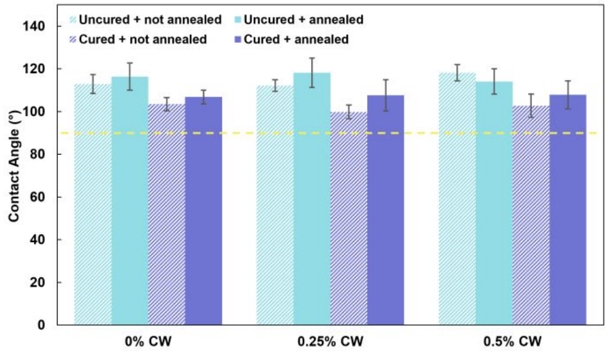

Figure S4. Water contact angles of coatings on paper. The yellow dotted line marks a contact angle of 90˚.

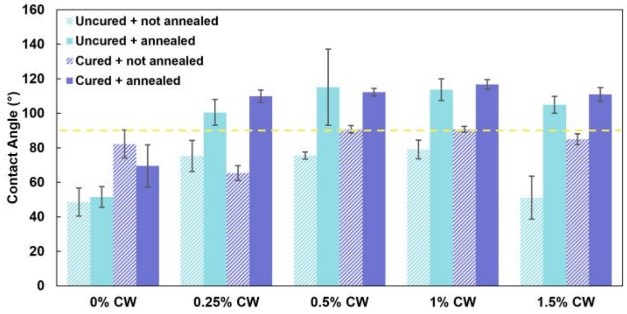

Figure S5. Water contact angle of coatings on Si wafers with CW content ranging from 0% to 1.5%. The yellow dotted line marks a contact angle of 90˚.

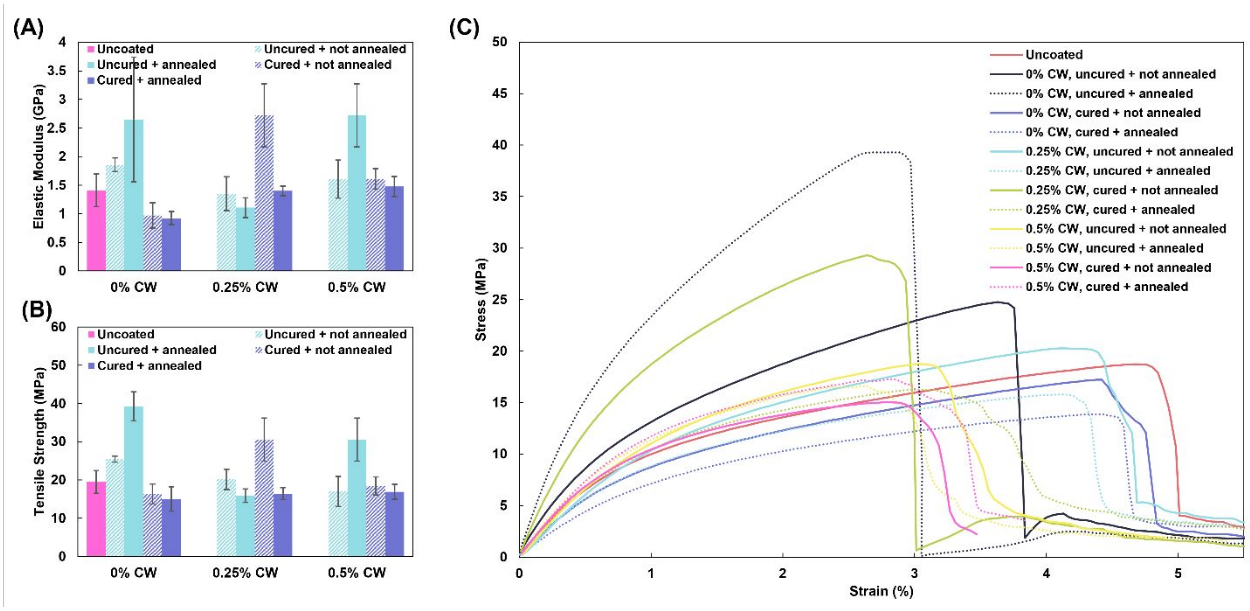

Figure S6. (A) Elastic modulus of uncoated and coated paper, (B) tensile strength of uncoated and coated paper, and (C) representative stress/strain curves from dynamic mechanical analysis of uncoated and coated paper.

Table S1. Coat weight and weight gain of coatings on paper.

Coating	Coat Weight (gsm)	Weight Gain (%)
0% CW, uncured + not annealed	5.1 ± 0.9	4.6 ± 0.8
0% CW, uncured + annealed	3.7 ± 2.4	3.4 ± 2.2
0% CW, cured + not annealed	4.6 ± 2.9	4.2 ± 2.6
0% CW, cured + annealed	2.8 ± 2.4	2.5 ± 2.1
0.25% CW, uncured + not annealed	6.9 ± 3.4	6.2 ± 3.1
0.25% CW, uncured + annealed	5.4 ± 2.8	4.9 ± 2.6
0.25% CW, cured + not annealed	1.6 ± 1.8	1.5 ± 1.7
0.25% CW, cured + annealed	2.4 ± 3.4	2.1 ± 3.1
0.5% CW, uncured + not annealed	7.3 ± 2.8	6.6 ± 2.5
0.5% CW, uncured + annealed	7.3 ± 1.9	6.6 ± 1.7
0.5% CW, cured + not annealed	–0.4 ± 1.2[a]	–0.3 ± 1.1[a]
0.5% CW, cured + annealed	4.5 ± 2.0	4.0 ± 1.8

[a]Negative values are attributed to negligible weight gain and density variation of heterogeneous paper.

Table S2. Water response and water vapor barrier properties of uncoated and coated paper.

Coating	Water Vapor Transmission Rate (g m^{-2} day^{-1})	$Cobb_{60}$ Water Absorptiveness (g m^{-2})	Water contact angle on paper substrate (°)	Water contact angle on Si wafer substrate (°)
N/A (Uncoated paper)	345.7 ± 4.7	131.6 ± 6.0	~0	N/A
0% CW, uncured + not annealed	334.0 ± 10.7	42.0 ± 10.2	112.9 ± 4.4	48.5 ± 8.2
0% CW, uncured + annealed	320.5 ± 34.2	40.7 ± 9.4	116.4 ± 6.3	51.5 ± 6.0
0% CW, cured + not annealed	299.2 ± 17.7	82.6 ± 21.0	103.4 ± 3.1	82.2 ± 8.1
0% CW, cured + annealed	303.8 ± 19.2	92.8 ± 20.4	106.8 ± 3.2	69.5 ± 12.3
0.25% CW, uncured + not annealed	294.8 ± 29.8	29.5 ± 0.8	112.2 ± 2.7	75.2 ± 9.0
0.25% CW, uncured + annealed	303.5 ± 34.0	29.1 ± 1.8	118.1 ± 6.9	100.5 ± 7.5
0.25% CW, cured + not annealed	311.0 ± 14.5	56.0 ± 6.0	99.8 ± 3.2	65.3 ± 4.4
0.25% CW, cured + annealed	260.7 ± 4.0	50.1 ± 3.7	107.6 ± 7.4	109.8 ± 3.6
0.5% CW, uncured + not annealed	329.7 ± 5.6	32.6 ± 2.9	118.1 ± 3.8	75.5 ± 2.1
0.5% CW, uncured + annealed	300.5 ± 31.6	31.3 ± 5.3	114.1 ± 5.9	115.1 ± 22.1
0.5% CW, cured + not annealed	309.3 ± 0.5	52.3 ± 9.5	102.7 ± 5.5	90.7 ± 2.1
0.5% CW, cured + annealed	304.0 ± 14.4	43.2 ± 8.7	107.8 ± 6.5	112.2 ± 2.3

Table S3. Oxygen and grease barrier properties of uncoated and coated paper.

Coating	Oxygen Transmission Rate ($cm^3\ m^{-2}\ day^{-1}$)	$Cobb_{60}$ Oil Absorptiveness ($g\ m^{-2}$)	Kit Rating
N/A (Uncoated paper)	12,500,000	94.1 ± 8.8	~0
0% CW, uncured + not annealed	125,000	51.2 ± 10.5	4.0 ± 1.2
0% CW, uncured + annealed	228,000	40.6 ± 9.6	3.0 ± 1.0
0% CW, cured + not annealed	130,000	48.9 ± 10.4	1.6 ± 0.5
0% CW, cured + annealed	230,000	48.3 ± 5.7	3.1 ± 0.8
0.25% CW, uncured + not annealed	185,000	51.0 ± 17.8	4.6 ± 0.9
0.25% CW, uncured + annealed	155,000	53.4 ± 8.1	3.4 ± 0.9
0.25% CW, cured + not annealed	355,000	47.9 ± 12.0	2.4 ± 0.5
0.25% CW, cured + annealed	617,500	47.8 ± 12.3	2.8 ± 0.8
0.5% CW, uncured + not annealed	Not tested	59.7 ± 12.8	3.6 ± 0.9
0.5% CW, uncured + annealed	Not tested	59.1 ± 11.6	3.6 ± 1.1
0.5% CW, cured + not annealed	Not tested	67.2 ± 17.4	2.6 ± 0.5
0.5% CW, cured + annealed	Not tested	62.5 ± 8.8N/	3.2 ± 1.1
